# Experimental Swine Models for Vascularized Composite Allotransplantation and Immunosuppression: A Systematic Review and Case Report of a Novel Heterotopic Hemifacial Swine Model

**DOI:** 10.3389/ti.2025.14520

**Published:** 2025-07-29

**Authors:** Leonard Knoedler, Felix J. Klimitz, Lioba Huelsboemer, Tobias Niederegger, Thomas Schaschinger, Samuel Knoedler, Sam Boroumand, Stav Brown, Bohdan Pomahac, Martin Kauke-Navarro

**Affiliations:** ^1^ Charité – Universitätsmedizin Berlin, Corporate Member of Freie Universität Berlin, Humboldt-Universität zu Berlin, and Berlin Institute of Health, Department of Oral and Maxillofacial Surgery, Berlin, Germany; ^2^Department of Surgery, Division of Plastic Surgery, Yale School of Medicine, New Haven, CT, United States; ^3^Department of Hand-, Plastic and Reconstructive Surgery, Microsurgery, Burn Trauma Center, BG Trauma Center Ludwigshafen, University of Heidelberg, Ludwigshafen, Germany

**Keywords:** swine models, vascularized composite allotransplantation, immunosuppression, graft rejection, preclinical research

## Abstract

Lifelong immunosuppression is necessary to prevent rejection in vascularized composite allotransplantation (VCA). Animal models play a pivotal role in developing innovative immunosuppressive strategies. This systematic review and case report focuses on the most impactful swine VCA models while offering insights gained from the Yale Swine Allotransplantation Vascularized Experiment (Y-SAVE). 22 studies on swine VCA models were included. Key swine breeds included SLA-matched and mismatched MGH miniature swine, Yucatan miniature swine, and outbred domestic swine. Transplantation models varied, with 10 (45%) using osteomyocutaneous flaps and only 2 (9%) involving hemifacial flaps. While 16 (73%) studies utilized heterotopic models, 5 (23%) relied on orthotopic models. Novel strategies such as preconditioning and localized drug delivery emerged, alongside immunosuppression regimens combining tacrolimus with experimental therapies. We further introduced a modified heterotopic hemiface VCA model, demonstrating its feasibility for studying immune dynamics in facial transplants while preserving oral function and enabling serial skin and mucosal biopsies. Overall, our review highlights a notable gap in models that specifically investigate facial VCAs. Given the unique immunological environment of facial allografts, models such as the heterotopic hemiface transplant may offer critical insights into immune mechanisms and may provide a platform for refining targeted immunosuppressive strategies.

## Graphical Abstract

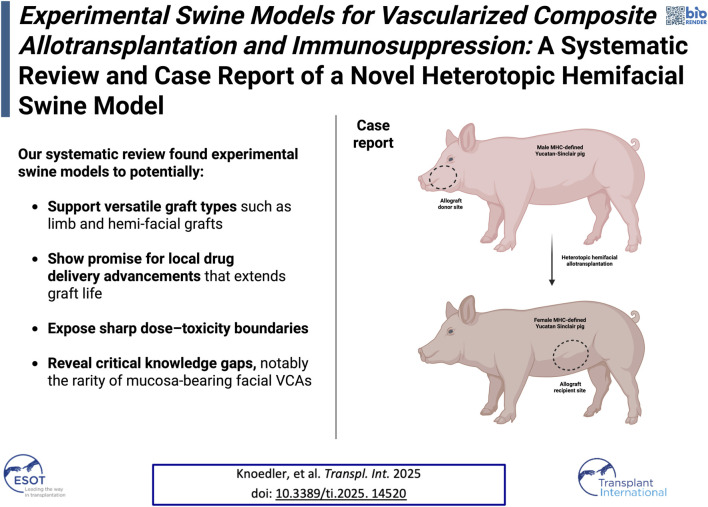

## Introduction

Vascularized composite allotransplantation (VCA) represents an innovative surgical approach to restore form and function of patients with devastating deformities [1–5]. Moving beyond the boundaries of conventional reconstructive approaches (such as autologous free tissue transfer and local tissue re-arrangement), VCA surgery has emerged as a valuable therapeutic option for patients with severe injuries or irreversible tissue loss [6, 7]. Over the past decades, a growing number of VCAs have been performed, yielding positive short- and long-term outcomes [8, 9]. VCAs include different tissues such as skin, mucosa, muscle, bone, lymphatics, vasculature and nerves. The inclusion of different tissue types with varying antigenicity is associated with a strong immune response by the recipient [10, 11]. In particular, epithelial surface tissues such as the skin and mucosa seem to be the primary targets of alloreactivity, mainly via a lymphocyte mediated adaptive immune response [12, 13].

Graft rejection (both acute and chronic) persists as the main barrier in VCA surgery, limiting its more widespread application. To control allograft rejection, recipients are administered lifelong immunosuppressive (IS) regimens, typically consisting of tacrolimus, mycophenolate mofetil (MMF) and prednisolone [14, 15]. Such immunosuppressants have a variety of side effects, for instance nephrotoxicity and an increased risk of malignancy and opportunistic infections. Despite high intensity IS protocols, ∼85% of VCA recipients still experience rejection episodes during the first year post-transplant and continue to reject almost annually, underlining the insufficiency of current immunomodulating strategies in VCA surgery [13, 16]. Besides acute graft rejection, patients face additional challenges such as chronic rejection which may lead to loss of functon and structure of the graft over time [17–19].

Large animal models, particularly swine, are invaluable for investigating novel immunomodulatory strategies with potential applications in human VCA recipients [20]. However, there is a notable lack of comprehensive research consolidating the current knowledge of swine models in this field. This gap represents an untapped opportunity to enhance *in vivo* experimentation and accelerate the translation of findings from the laboratory to clinical practice. To address this, we systematically reviewed the existing literature on experimental swine models in VCA, examining their indications, strengths, and limitations. Additionally, we detail the planning and outcomes of the Yale Swine Allotransplantation Vascularized Experiment (Y-SAVE). This research aims to advance the refinement of swine models and address persistent challenges in VCA surgery, ultimately improving their utility and translatability.

## Methods

### Search Strategy

This systematic review was conducted according to the Preferred Reporting Items for Systematic Reviews and Meta-Analysis (PRISMA) guidelines [21]. The MEDLINE database (PubMed) and Google Scholar were queried for relevant articles published until November 13th, 2024. All studies had to be written in English. Only articles presenting original data were included. Only articles discussing experimental swine models for vascularized composite allotransplantation and immunosuppression were eligible.

### Data Extraction and Quality and Bias Assessment

The search strategy for PubMed/MEDLINE and Google Scholar was developed (Supplementary Table S1). Two reviewers (LK, FK) independently screened all articles by title and abstract. Articles were subsequently analyzed in greater depth through full-text assessment to determine eligibility. Any disagreements regarding the inclusion of individual studies were resolved through consultation with a third author (MK). For included articles, citation searching was carried out on Google Scholar. Data extraction was performed independently by two authors (LK, FK) to ensure accuracy and consistency. During the blinded, dual-review process, we extracted the following variables for each study included: Digital Object Identifier (DOI), first author, study title, year of publication, region of publication, number of animals, mean age, gender, follow-up (mean and range), and the specifics of performed procedures. To evaluate the quality and risk of bias of the included studies, the SYRCLE risk of bias (RoB) tool for animal studies was employed [22]. The detailed risk of bias assessments for all studies are presented in Supplementary Table S2.

### Case Report

To complement the findings of this systematic review, we included a representative case report describing a novel heterotopic hemifacial VCA model in swine. This model was developed in response to gaps identified in the literature, particularly the lack of large-animal models incorporating facial tissue and permitting mucosal assessment. The case report provides detailed procedural insights and demonstrates the feasibility of serial mucosal and skin biopsies in a controlled, minimally invasive manner. Its inclusion offers practical context and supports the translational relevance of emerging strategies for immune monitoring in facial allotransplantation.

## Results

**FIGURE 1:**
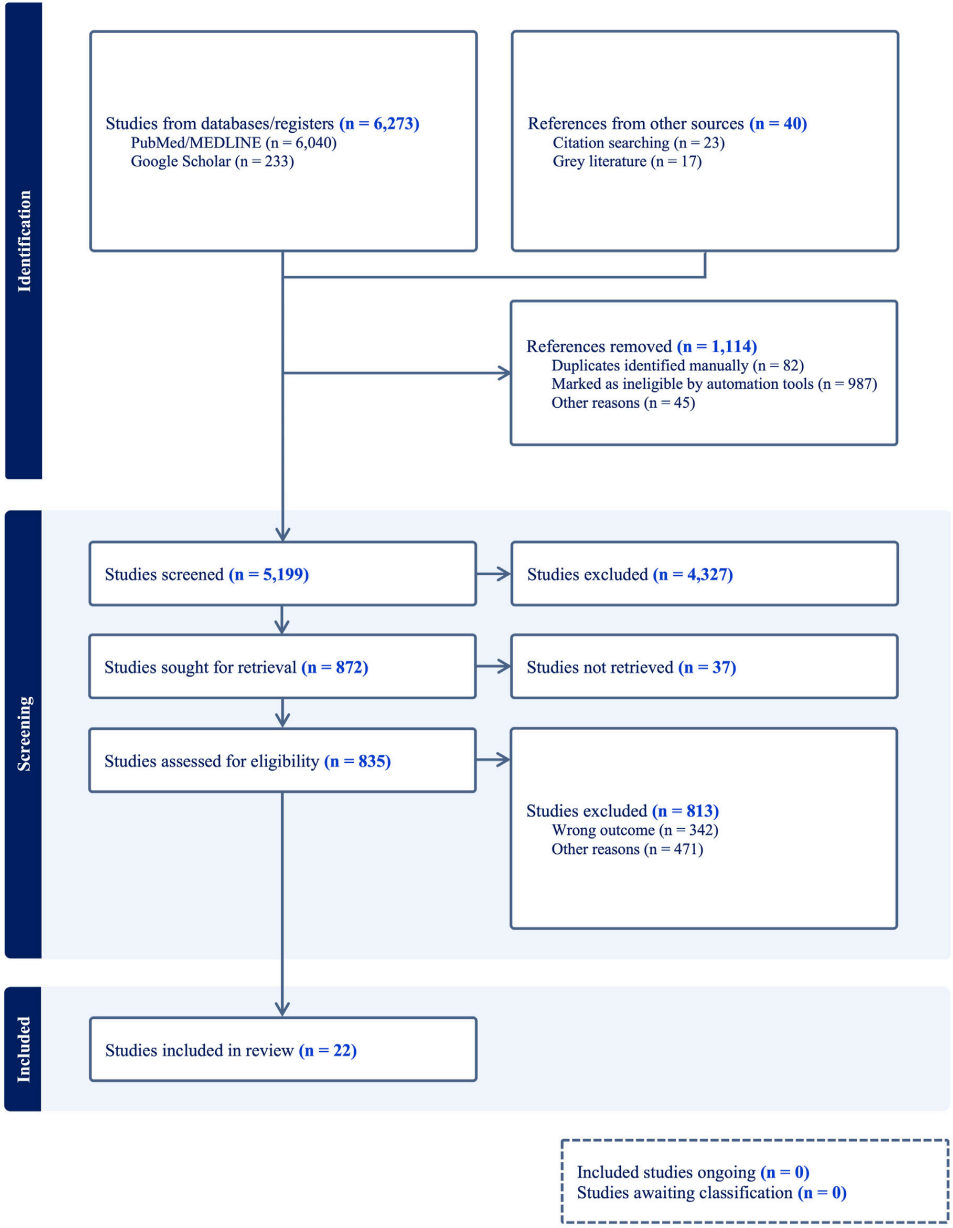
PRISMA 2020 Flowchart of the conducted methodology.

After full-text analysis, a total of 22 eligible studies were included in the qualitative synthesis. A PRISMA flowchart of study identification, screening, and inclusion is presented in Figure 1.

### Swine Models

We identified various swine breeds that were employed to examine VCA. The primary models included MGH miniature swine [23–26], Yucatan miniature swine [27, 28], and outbred domestic swine [29–31], which were selected for their genetic similarities to human immunologic responses. In particular, MGH miniature swine and Yucatan miniature swine were frequently chosen for their manageable size and robust immunological profiles. Other studies utilized outbred Yorkshire swine and Swiss Landrace pigs [32–34], adding diversity in immune response due to genetic variability, which allowed for a comprehensive analysis of transplantation outcomes across multiple immune phenotypes. Details are reported in Table 1.

**TABLE 1:** Overview of studies on Experimental Swine VCA models.

Author and year	Study design	Animals used	Transplant model	Heterotopic/orthotopic	Facial VCA (yes/no)	Tissue type	Donor	Recipient
Barone et al. [23]	*In vitro*	Complete MHC mismatched MGH miniature swine	Gracilis VCA transplanted to the cervical region	Heterotopic	No	Myocutaneous	MGH miniature swine	MGH miniature swine
Berkane et al. [33]	*Ex vivo*	Female Yorkshire pigs	Bilateral partial hindlimb VCA	N/A	No	Osteomyocutaneous	Female Yorkshire pigs	N/A
Blades et al. [43]	*In vivo*	Sinclair and Yucatan pigs	VRAM flap transplanted to the neck region	Heterotopic	No	Myocutaneous	Sinclair pigs	Yucatan pigs
Elgendy et al. [26]	*In vitro*	SLA- mismatched MGH miniature swines	VRAM flap transplanted to dorso-lateral neck region	Heterotopic	No	Myocutaneous	MGH miniature swine	MGH miniature swine
Fries et al. [35]	*In vivo*	SH- mismatched miniature swine	Radio-ulnar forelimb VCA	Orthotopic	No	Osteomyocutaneous	SH- mismatched Yucatan miniature pigs	SH- mismatched Yucatan miniature pigs with four SLA-HS
Ibrahim et al. [36]	*In vivo*	MHC-defined inbred MGH miniature swine	Hind limb VCA transplanted to subcutaneous abdominal wall pockets	Heterotopic	No	Osteomyocutaneous	Male MGH miniature swine	Female miniature swine
Kim et al. [25]	*In vivo*	Fully MHC mismatched MGH miniature swine	Hind-limb VCA model	Heterotopic	No	N/A	MGH mini-swine	MGH mini-swine
Kotsougiani et al. [28]	*In vivo*	Yucatan miniature pig	Tibial defect VCA model	Orthotopic	No	Osteomyocutaneous	Yucatan mini pig tibia (SLA- and blood type compatibility)	Yucatan mini pig tibia, age and size matched (SLA- and blood type compatibility)
Kuo et al. [29]	*In vitro*	Outbred miniature swine (genotypes: GPI-BB and PGD-AA)	Hind limb VCA transplanted to subcutaneous abdominal wall pockets	Heterotopic	No	Osteomyocutaneous	Outbred miniature swine (lan-yu strain; age 3 months; weight 12–20 kg)	Outbred miniature swine (lan-yu strain; age 3 months; weight 12–20 kg)
Kuo et al. [29]	*In vivo*	Outbred miniature swine (Lan-Yu and Hwa-Ban strains)	Hemi-facial flap (skin, muscle, ear cartilage, nerve, parotid gland, surrounding tissue)	Orthotopic	Yes	Chondromyocutaneous	Lan-Yu and Hwa-Ban strain	Lan-Yu strain
Kuo et al. [30]	*In vitro*	Outbred miniature swine	Hind limb VCA transplanted to subcutaneous abdominal wall pockets	Heterotopic	No	Osteomyocutaneous	Outbred miniature swine lan-yu strain; age 3 months; weight 12–20 kg	Outbred miniature swine lan-yu strain; age 3 months; weight 12–20 kg
Kuo et al. [31]	*Ex vivo*	Outbred miniature swine	Hind limb VCA transplanted to subcutaneous abdominal wall pockets	Heterotopic	No	Osteomyocutaneous	Female outbred miniature swine	Male outbred miniature swine
Leonard et al. [24]	*In vitro*	MGH miniature swine	Hind limb VCA transplanted to neck or abdominal wall region	Heterotopic	No	Fasciocutaneous	MGH miniature swine with PAA-positive SLA	MGH miniature Swine with PAA-negative SLA
Mathes et al. [32]	*In utero* and *in vitro*	MGH miniature swine and outbred Yorkshire sows and boars	Hind limb VCA transplanted to subcutaneous abdominal wall pockets	Heterotopic	No	Osteomyocutaneous	SLA homozygous MGH miniature swine	Outbred Yorkshire sow and boar fetuses (negative for SLA class I^c^)
Park et al. [44]	*In vivo*	Domestic swine	Hemi-facial flap (skin, mucosa, subcutaneous fat tissue, ear, maxilla and mandibular bone)	Orthotopic	Yes	Osteochondrocutaneous	Domestic swine	Domestic swine
Shanmugarajah et al. [38]	*In vitro*	Miniature MGH swine model	Hind limb VCA transplanted to the neck region	Heterotopic	No	Fasciocutaneous	HC miniature swine model (SLA^gg^ class I^c^/ii^d^)	HC miniature swine model (SLA^cc^ class I^c^/ii^c^)
Tratnig-Frankl et al. [39]	*In vivo*/*ex vivo*	MHC-defined miniature swine	Gracilis VCA model	Orthotopic	No	Myocutaneous	MHC-defined miniature swine	MHC-defined miniature swine (group 1: class I and class II match; group 2: class I and class II missmatch)
Wachtman et al. [27]	*In vitro*	Yucatan miniature swine	Hind limb VCA transplanted to subcutaneous abdominal wall pockets	Heterotopic	No	Osteomyocutaneous	Yucatan miniature swine	Yucatan miniature swine
Waldner et al. [40]	*In vivo*	Partially inbred SLA–mismatched miniature swine (homozygous HC alleles)	VRAM flap transplanted to the neck region	Heterotopic	No	Myocutaneous	Miniature swine (hetero- and homozygous for HC; 2-3 months old; weight between 10 and 20 kg; full SLA mismatch	Miniature swine, (hetero- and homozygous for HC; 3-5 months old; weight between 20 and 30 kg; full SLA mismatch
Wang et al. [45]	*Ex vivo*	Yorkshire swines (SLA-mismatch in one)	Gracilis VCA transplanted to the neck region	Heterotopic	No	Myocutaneous	Yorkshire swine	Yorkshire swine
Wu et al. [42]	*In vivo*	SLA- mismatch swine	Gracilis VCA model	Heterotopic	No	Myocutaneous	Swine with single SLA mismatch	Swine with single SLA mismatch
Zhang et al. [34]	*In vivo*	MHC-mismatched Swiss landrace pigs	Knee VCA transplanted to subcutaneous abdominal wall pockets	Heterotopic	No	Osteomyocutaneous	Swiss landrace pigs (MHC-mismatched; aged 11–14 weeks)	Swiss landrace pigs (MHC-mismatched)

MGH, Massachusetts General Hospital; SLA, Swine leukocyte antigen; HC, histocompatibility complex; MHC, major histocompatibility complex; VCA, vascularized composite allotransplant; SH, single haplotype; PAA, pig allelic antigen; CS, Cold Storage; TAC, tacrolimus; MMF, Mycophenolate Mofetil; MPDN, Methylprednisolone; CXA, cyclosporine A; CD3-IT, CD3-Immunotoxin; (CTLA4-Ig), cytotoxic T-lymphocyte antigen 4 immunoglobulin; POD, postoperative day; AR, acute rejection; DSAs, donor specific antibodies; N/A, not applicable; TGMS, triglycerol monostearate; VRAM, vertical rectus abdominus myocutaneous flap, ASC, adipose-derived stem cell; AV, arteriovenous.

### Transplant Models and Interventions

Various transplantation models were utilized, including heterotopic hind-limb transplantation, gracilis myocutaneous flaps, vertical rectus abdominis myocutaneous (VRAM) flaps, osteomyocutaneous flaps, partial hindlimb models, forelimb models, and tibial VCA. Of the n = 22 studies included, n = 16 (73%) applied heterotopic VCA models, while n = 5 (23%) used orthotopic models [23, 24, 26–32, 34–44]. Notably, n = 1 study (5%) did not categorize the approach as either heterotopic or orthotopic and n = 10 (45%) studies included osteomyocutaneous VCAs [27, 28, 30–37]. Furthermore, n = 2 (9%) studies performed hemi-facial VCAs, with n = 1 involving transplantation of the maxillo-mandibular complex [29, 44]. Lastly, assessment of mucosal tissue was reported in n = 1 (5%) of studies included.

Orthotopic models were employed by fewer authors. Fries et al. utilized an orthotopic mismatched porcine forelimb VCA model in SH-mismatched Yucatan miniature pigs [35]. Kotsougiani et al. implemented an orthotopic tibial defect VCA model in SLA- and blood type-compatible Yucatan miniature pigs [28]. Tratnig-Frankl et al. used an orthotopic gracilis myocutaneous free flap model in MHC-defined miniature swine to assess the impact of antioxidant therapies on graft survival [39]. Interestingly, Kuo et al. employed an orthotopic hemi-facial chondromyocutaneous flap, including skin, muscle, ear cartilage, and parotid gland in Lan-Yu miniature swine to study rejection dynamics and Park et al. utilized an orthotopic hemi-facial osteochondrocutaneous flap, incorporating skin, mucosa, subcutaneous tissue, ear cartilage and the maxillo-mandibular complex in domestic swine to investigate vascular and skeletal fixation techniques [29, 44]. More information is provided in Table 1.

### Immunosuppressive Strategies

Multiple immunosuppressive strategies were employed across different VCA models. These approaches included total body irradiation (TBI), thymic irradiation, T-cell depletion, bone marrow transplantation (BMT), and targeted drug therapies such as tacrolimus (TAC), cyclosporine A (CXA), mycophenolate mofetil (MMF), and mTOR inhibitors (e.g., rapamycin). Outcomes varied based on the immunosuppressive regimen and dosages used.

Starting with Barone et al., the authors combined low-dose total body irradiation (100cGy 2 days prior to surgery or 200cGy divided in 2 × 100 cGy doses on preoperative day 2 and 3), T-cell depletion with CD3 immunotoxin (0.05 mg/kg i.v., twice daily from preoperative day 4 to day 0), CXA (target level 400–800 ng/mL), and donor bone marrow cell infusion (7.8 × 10^8^ to 4 × 10^9^ cells/kg of recipient body weight) alongside VCA to achieve mixed chimerism, though this was insufficient for complete tolerance induction [23]. Ibrahim et al. employed short-term TAC monotherapy (target levels of 10–15 ng/mL) in a VCA model with intact vascularized bone marrow, demonstrating long-term graft survival with viable vascularized bone marrow and successful immune monitoring [36]. Kim et al. utilized a 30-day TAC course combined with adipose-derived stem cell (ASC) therapy (1.0 × 10^6^ cells/kg administered intravenously on postoperative day (POD) 7), achieving rejection-free survival for over 200 days while significantly upregulating T-regulatory cells and donor-specific unresponsiveness. Elgendy et al. compared the efficacy of mTOR inhibitors, finding that TAC (0.1–0.125 mg/kg) significantly delayed acute rejection (grade I AR on POD 30 and grade IV AR on POD 74) compared to rapamycin (0.02–0.2 mg/kg), which led to rapid rejection (grade IV AR by POD 17-20) [26]. Conversely, Fries et al. employed low-dose TAC (49 mg) administered via an enzyme-responsive hydrogel platform, which prolonged graft survival, whereas high doses (91 mg) caused poor tolerance and complications such as weight loss and pancreatitis [35]. Kotsougiani et al. used a combination of TAC (target levels of 5–30 ng/mL), MMF (target levels of 1–3.5 ng/mL), and methylprednisolone (tapered to 0.1 mL for maintenance), achieving graft survival and enhancing vascular remodeling without rejection during the 4-month follow-up [28]. Meanwhile, Kuo et al. combined irradiation, BMT, and CXA with mesenchymal stem cells (MSCs) in variying dosages, resulting in significantly prolonged graft survival and reduced acute rejection. Here, increased regulatory T-cell populations (CD4^+^/CD25^+^ and CD4^+^/FoxP3^+^) were found [30, 31, 37]. Leonard et al. applied 100 cGy total body irradiation, T-cell depletion with CD3 immunotoxin (50 μg/kg), and hematopoietic cell transplantation (15 × 10^9^ cells/kg), achieving stable mixed chimerism and long-term graft acceptance without signs of rejection up to POD 504 [24]. Mathes et al. pioneered an *in utero* bone marrow transplantation approach, achieving multilineage macrochimerism and donor-specific tolerance without prolonged post-transplant immunosuppression. The authors relied on CXA (target levels of 400–800 ng/mL) post-bone marrow infusion (2 × 10^9^ cells/kg) to maintain donor-specific tolerance, demonstrating effective rejection prevention in chimeric animals [32]. Furthermore, Shanmugarajah et al. utilized T-cell depletion with CD3 immunotoxin (50 μg/kg), 100 cGy TBI, and a 45-day CXA regimen (target levels of 400–800 ng/mL) to achieve immune tolerance in MHC class II mismatched chimeras, although MHC class I mismatched animals experienced rejection [38]. Meanwhile, Kuo et al. demonstrated that CXA delayed rejection from POD 7 to 28 in untreated controls to POD 38 to 49 in their hemi-facial VCA model [29]. Additionally, strategies explored by Wu et al. focused on enzyme-responsive and TAC-eluting hydrogels. The authors demonstrated prolonged survival using hydrogel-administered TAC (28 mg/4 cc and 49 mg/4 cc), effectively delaying grade IV AR to POD 20 and 28 [42].

Overall, five studies did not administer immunosuppressive therapies. For instance, Blades et al. observed flap rejection between POD 5 and 9 without immunosuppressive treatment and Park et al. by POD 14 to 18 [43, 44]. Tratnig-Frankl et al. and Wang et al. did not administer immunosuppression to avoid skewing of rejection periods in novel treatment approaches. Tratnig-Frankl et al. investigated H_2_S and NaI treatments but observed no significant differences in graft survival or immunological outcomes compared to saline controls [39]. In Wang et al. the experimental group received hyperoxygenated University of Wisconsin solution and showed significantly later onset of grade 1 AR, compared to the control group [45]. Lastly, in Berkane et al. the study protocol did not foresee immunosuppression [33]. Further information can be found in Table 2.

**TABLE 2:** Overview of Interventions, Immunosuppressive Strategies and Outcomes of studies included.

Author and year	Interventions	Immunosuppresion	Outcomes	Complications
Barone et al. [23]	Bone marrow transplantation	Low-dose total body irradiation (100cGy 2 days prior to surgery or 200cGy divided in 2 × 100 cGy doses on preoperative day 2 and 3), T-cell depletion wirh CD3-IT (0.05 mg/kg), CXA (target level 400–800 ng/mL), donor bone marrow cells (7.8 × 10^8^ to 4 × 10^9^ cells/kg of recipient body weight)	Bone marrow infusion led to better clinical outcomes; chimerism detected but insufficient for tolerance	Mixed chimerism after bone marrow transplantat; VCA appeared insufficient for tolerance induction
Berkane et al. [33]	Two study groups: supercooling intervention group and cold storage control group undergoing subsequent normothermic machine perfusion	No immunosuppressive therapy used	Supercooled VCAs restored vascular flow and had lower resistance during machine perfusion	N/A
Blades et al., 2024 [43]	Investigation of possible surgical complications	No immunosuppressive therapy used	All flaps survived initially, with adequate perfusion for 4 days. Flap rejection occurred between POD 5 and POD 9 in all animals	Minimal erythema observed post-transplant, no surgery-related deaths or infections
Elgendy et al. [26]	Treatment with Co-stimulation blockade and mTOR inhibitor, with or without preceding short-term calcineurin inhibitor therapy	mTOR inhibitor (rapamycin [0.02–0.2 mg/kg] or tacrolimus [0.1–0.125 mg/kg])	TAC delayed AR (grade-I AR on POD 30, grade-IV on POD 74); rapid rejection with rapamycin (grade-I AR by POD 2 and 7, grade-IV AR by POD 17–20)	Rejection of allograft, erythema, severe necrotizing T cell mediated rejection with deep dermal arterial thrombosis
Fries et al. [35]	Tacrolimus eluting hydrogel implants with various concentrations (91 mg, high dose/49 mg, low dose)	Graft-implanted enzyme-responsive, TAC eluting hydrogel platform	Low-dose TAC prolonged survival; high-dose TAC caused poor tolerance (grade IV AR from POD 56–93)	High dose TAC group: one sample excluded due to flap failure on POD 1; four animals showed poor feeding and weight loss, requiring early euthanasia; four animals from high dose TAC group developed pancreatitis
Ibrahim et al. [36]	Development of novel tranlational VCA research model	Short-term tacrolimus monotherapy (target levels of 10–15 ng/mL) with or without bone marrow infusion	Long-term graft survival (>150 days) with viable vascularized bone marrow; successful immune monitoring	Venous thrombus in one case resolved by reanastomosis, no graft-versus-host disease
Kim et al. [25]	Treatment with tacrolimus for 30 days and ASC therapy (donor-derived ASCs [1.0 × 10^6 cells/kg])	TAC, ASC-therapy	Adipose-derived stem cells demonstrated grade IV AR on POD 119 and rejection-free survival over POD 200 as well as upregulated T-regulatory cells	The control group reached Banff grade 4 acute rejection by an average of 7.5 days after transplantation. Allografts treated with ASCs demonstrated grade 4 rejection on day 119
Kotsougiani et al. [28]	AV-bundle implantation in tibial allotransplant	TAC (target levels of 5–30 ng/mL), MMF (target levels of 1–3.5 ng/mL), MPDN (tapered to 0.1 mL)	Micro-CT showed bone formation and remodeling at the distal allograft junction; allograft survived without any healing problems or limited hindlimb perfusion during the 4-month follow-up	N/A
Kuo et al. [29]	Treatment with various dosages of mesenchymal stem cells, CXA, bone marrow transplantation and irradiation	Irradiation, bone marrow trnsplantation and CXA	Mesenchymal cells extended graft survival, combined CXA and stem cells showed significantly better survival, allografts with CXA exhibited delayed AR, examination of bromodeoxyuridine-labeled mesenchymal stem cells revealed donor mesenchymal stem cells engraftment into the recipient and donor skin	Graft-versus-host disease evident in CXA group
Kuo et al. [29]	Comparison of rejection in untreated, control and CXA-treatment groups	CXA in treatment group, untreated and control: N/A	100% survival rate, CXA treatment delayed flap rejection significantly (POD 38-49), no significant difference in rejection signs in allo-cartilage	Swelling for 2 weeks (postoperative saliva gland hypersecretion), control group: progressive rejection by POD 7-28, lymphoid gland tissue and skin were susceptible to early rejection
Kuo et al. [30]	Various combinations of mesenchymal stem cells cyclosporine or irradiation	Mesenchymal stem cells, CXA, irradiation	Mesenchymal stem cells with irradiation and CXA: significantly increased allograft survival compared with other groups (>120 days; p < 0.01); histology showed lowest degree of AR in grafted skin and interstitial muscle layers in mesenchymal stem cell/irradiation/CXA group; significant increase in percentage of CD4+/CD25+ and CD4+/FoxP3+ T in the mesenchymal stem cell/irradiation/CXA group	Rejection episodes
Kuo et al. [31]	Various dosages of ASCs, tacrolimus or irradiation	TAC, irradiation	Multiple injections of adipose-derived stem cells, irradiation and TAC increased allograft survival significantly	Lymphocyte infiltration in the alloskin and interstitial muscle layers of treatment group
Leonard et al. [24]	Stem cell transfusion	100 cGy irradiation, T cell depletion with CD3-IT (50 μg/kg), hematopoietic cell transplantation (15 × 10^9^ cells/kg)	Following withdrawal of immunosuppression both VCAs transplanted into stable chimeras Recipients of hematopoietic cell transplantation displayed no clinical signs of AR up to POD 504	Two animals developed skin graft versus host disease
Mathes et al. [32]	Treatment with CXA and bone marrow transplantation (2 × 10^9^ cells/kg)	CXA (target levels of 400–800 ng/mL)	Donor cell engraftment and multilineage macro chimerism after *in utero* transplantation of adult bone marrow cells, and chimeric animals were unresponsive to donor antigens *in vitro*; both control VCAs rejected by POD 21; chimeric animals accepted VCAs (no DSAs or alloreactivity)	All grafts demonstrated some mild lymphocytic infiltration at the day 7 biopsy. All of the animals developed a severe dermal perivascular lymphocytic infiltration with scattered eosinophils and went on to reject their donor skin grafts
Park et al. [44]	Vascular anastomosis of the carotid artery and jugular vein, fixation of the maxillo-mandibular complex with titanium plates	No immunosuppressive therapy used	Successful transplant without early arterial or venous insufficiency, acute rejection from POD 7-8 onwards	Acute rejection POD 7-8, pink discoloration, edema, erythematous papule with flap necrosis on POD 14–18
Shanmugarajah et al. [38]	Hematopoietic stem cell transplantant, irradiation	T cell depletion with CD3-IT (50 μg/kg), 100 cGy TBI and 45 days of CXA (target levels of 400–800 ng/mL)	HC class II–mismatched chimeras were tolerant of VCAs; HC class I–mismatched animals rejected VCA skin, (infiltration of CD8^+^ lymphocytes)	One HC class II mismatched model displayed clinical features of chronic graft versus host disease (euthanized on POD 190)
Tratnig-Frankl et al. [39]	Treatment with either saline (control), sodium iodide (NaI), or hydrogen sulfide (H_2_S) injections	No postoperative immunosuppression	No effect of H_2_S or NaI treatment in comparison to NaCl in delaying AR, flap survival and histology revealed no significant differences between the groups	One technical failure occurred in the saline MISMATCH subgroup
Wachtman et al. [27]	Bone marrow infusion and irradiation	Total body (100 cGy) and thymic (700 cGy) irradiation, bone marrow infusion, tacrolimus (0.1 mg/kg/day), CTLA4-Ig (20 mg/kg)	Experimental groups rejected allografts (skin and muscle) on POD 5 to 30; skin and muscle histology in all long-term survivors were normal	Rejection episodes
Waldner et al. [40]	Investigation of VRAM flap applicability in VCA research	TAC, rapamycin, CTLA4-Ig	POD 5: all grafts demonstrated pale-pink skin color without edema; follow-up showed improved correlation between clinical appearance and progression of graft rejection in histology	Intraoperative cardiac arrest in one sample (death due to anesthesia); one recipient experienced flap loss due to venous compromise; Banff grade I AR with erythemous and edematous grafts
Wang et al. [45]	Treatment with sub-normothermic *ex-vivo* perfusion using hyper-oxygenated University of Wisconsin (UW) solution	No immunosuppressive therapy used	Experimental group showed significantly later onset of grade 1 AR at 13.7 days (SD = 0.52, p < 0.05); by POD 15 75% of the flaps showed no evidence of grade 4 AR	Rejection episodes
Wu et al. [42]	Treatment with various dosages (28 mg/4cc and 49 mg/4cc) of tacrolimus-eluting hydrogel injected into the donor flap	TAC-eluting hydrogel (28 mg/4cc and 49 mg/4cc)	TAC-eluting hydrogel prolonged graft survival in both groups (grade 4 AR on average by POD 20 and 28)	Rejection episodes
Zhang et al. [34]	Treatment with various combinations of TGMS and TAC	Locally administered TAC-loaded on-demand drug delivery system	Repeated intra-graft TGMS-TAC administrations prolong graft survival	Grade III-IV rejection

MGH, Massachusetts General Hospital; SLA, Swine leukocyte antigen; HC, histocompatibility complex; MHC, major histocompatibility complex; VCA, vascularized composite allotransplant; SH, single haplotype; PAA, pig allelic antigen; CS, Cold Storage; TAC, tacrolimus; MMF, Mycophenolate Mofetil; MPDN, Methylprednisolone; CXA, cyclosporine A; CD3-IT, CD3-Immunotoxin; (CTLA4-Ig), cytotoxic T-lymphocyte antigen 4 immunoglobulin; POD, postoperative day; AR, acute rejection; DSAs, donor specific antibodies; N/A, not applicable; TGMS, triglycerol monostearate; VRAM, vertical rectus abdominus myocutaneous flap; ASC, adipose-derived stem cell; AV, arteriovenous.

### Major Findings

In several models, immunosuppressive therapies and interventions significantly improved graft survival, with some protocols achieving long-term graft acceptance and reduced acute rejection (AR). For instance, Ibrahim et al. reported long-term graft survival exceeding 150 days with short-term TAC therapy and bone marrow infusion, highlighting the effectiveness of combining localized and systemic immunosuppression [36]. Similarly, Kim et al. observed prolonged rejection-free graft survival beyond 200 days using adipose-derived stem cell therapy combined with TAC, correlating the upregulation of regulatory T-cells (Tregs) with sustained graft tolerance [25]. Leonard et al. demonstrated stable chimerism and long-term graft survival up to 504 days following hematopoietic cell transplantation with irradiation and T-cell depletion, suggesting the importance of chimerism in inducing robust immune tolerance [24]. Additionally, Mathes et al. demonstrated that cyclosporine treatment combined with *in utero* bone marrow transplantation resulted in long-term chimeric stability and VCA acceptance, highlighting the effectiveness of early hematopoietic intervention [32].

Most studies reported significant success in prolonging graft survival with tailored immunosuppressive regimens. For example, Wang et al. observed a delay in acute rejection in flaps treated with systemic immunosuppression, with 75% of experimental flaps showing no rejection by day 15 [45]. Treatments combining stem cells with immunosuppressantsoften resulted in prolonged graft survival with reduced rejection rates, as seen in the work of Kuo et al. [30, 31, 37] In contrast, Elgendy et al. found that TAC significantly delayed AR compared to rapamycin, where rapid AR was observed [26]. Additionally, Fries et al. revealed that while low-dose TAC via enzyme-responsive hydrogels prolonged graft survival, high doses led to poor outcomes, including weight loss, pancreatitis, and early euthanasia [35]. Localized immunosuppressive delivery methods demonstrated promising results. Wu et al. utilized tacrolimus-eluting hydrogels, which effectively prolonged graft survival and delayed grade IV AR [42]. Similarly, Zhang et al. employed a localized tacrolimus-loaded drug delivery system, resulting in repeated intra-graft administration that significantly extended graft survival [34]. Kuo et al. found CXA to significantly delay rejection of hemi-facial flaps. Lastly, some treatments failed to demonstrate significant efficacy: Tratnig-Frankl et al. found no significant difference in graft survival or histological outcomes after antixodative therapy compared to controls [39]. Details are provided in Table 2.

### Complications

Complications varied with the immunosuppressive approach and transplant model. While many studies reported successful outcomes in terms of prolonged graft survival and delayed rejection, complications often arose from either the intervention protocols themselves or the adverse effects of immunosuppressive regimens. For example, Fries et al. reported weight loss, poor feeding, and pancreatitis in animals subjected to high-dose TAC therapy, with some requiring early euthanasia [35]. Similarly, Elgendy et al. noted rapid rejection with rapamycin treatment compared to TAC [26]. Furthermore, vascular complications such as venous thrombosis were reported. These complications were resolved through re-anastomosis without long-term graft loss [36]. Blades et al. observed flap rejection between POD 5 and 9, despite initial adequate perfusion, and noted minimal erythema as an early rejection marker [43]. Additionally, Tratnig-Frankl et al. reported a technical failure in one saline subgroup, emphasizing the role of surgical precision in preventing graft loss [39]. Systemic complications related to immunosuppression were also observed. Shanmugarajah et al. documented chronic graft-versus-host disease (GvHD) in one animal, requiring euthanasia by POD 190 [38]. Similarly, Kuo et al. noted GvHD in animals treated with cyclophosphamide and irradiation, indicating the risks associated with preconditioning regimens [30, 31, 37]. Additionally, Wachtman et al. reported histological evidence of graft rejection in skin and muscle components, despite long-term survival in other grafted tissues [27]. Surgical mortality due to anesthesia-related complications was also recorded. Waldner et al. described an intraoperative cardiac arrest in one recipient, as well as venous compromise leading to flap loss in another case [40]. Furthermore, complications such as poor perfusion, erythema, and edema were commonly cited as markers of early graft rejection, requiring close monitoring for timely intervention [40, 43]. More information is presented in Table 2.

In summary, these studies underline the potential of swine models to explore VCA and immunosuppressive strategies, revealing that combinations of traditional drugs like TAC and cyclosporine with novel agents or delivery systems can extend graft survival and reduce immune responses.

### Case Report

Based on these findings, we decided to perform a heterotopic hemiface transplantation procedure using an MHC-defined Yucatan Sinclair strain to establish a novel swine model consisting of heterotopic hemiface vascularized composite allotransplantation (VCA) to the groin area. This model specifically enables frequent biopsies due to the accessibility of the flap but more importantly including donor mucosa while allowing the receipient to ingeste, feeding, or persue related activities. In contrast, orthotopic transplantation risks confounding mucosal assessment, as the animal may chew on or manipulate the graft. This setup enables detailed analysis of immunological interactions at the skin and mucosa interfaces, thus providing valuable insights into tissue rejection dynamics and tolerance in a way that conventional graft sites may not accommodate as effectively. To conclude, the Yucatan Sinclair strain is furthermore well-suited for this purpose, given its immunologic compatibility in modeling human responses.

### Animals

**FIGURE 2:**
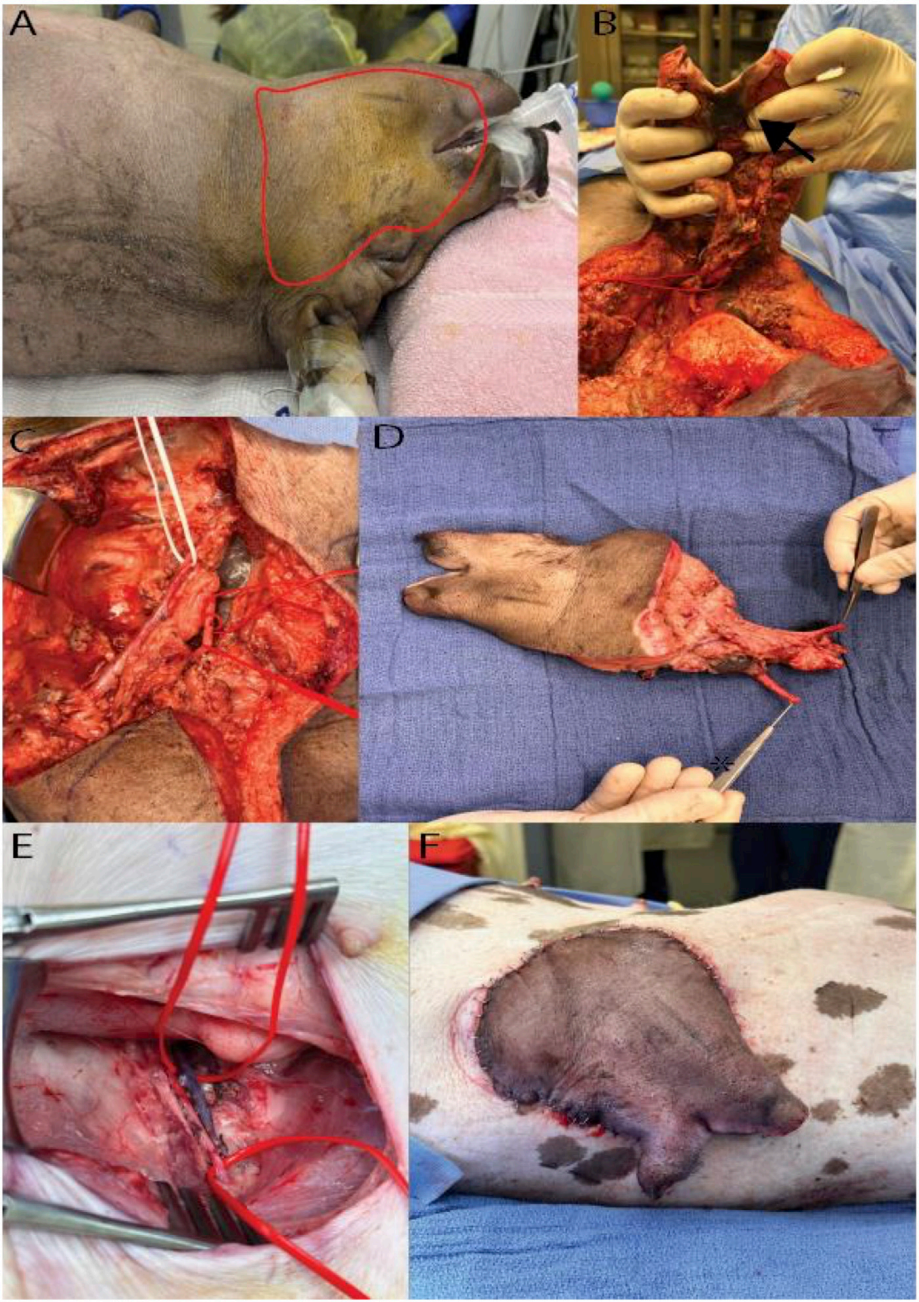
Hemifacial Heterotopic Transplant Model. **(A)** Outline of the hemifacial transplant. **(B)** Underside of the hemifacial graft after dissection, with an arrowhead marking the intraoral mucosa. **(C)** Demonstration of the vascular pedicle of the graft, with white vessel loop identifying the external jugular vein and red loops marking the common carotid artery. **(D)** Explanted hemifacial graft showing the vascular pedicle. **(E)** Dissected femoral vessels used for vascular anastomosis. **(F)** Hemifacial graft inset in the lateral abdominal wall post-transplantation.

A heterotopic hemiface vascularized composite allotransplantation to the groin area was performed from a male donor pig to a female recipient of MHC-defined Yucatan Sinclair strain. We performed the study following the *Guide for the Care and Use of Laboratory Animals* published by the National Institutes of Health [46]. Experiments were conducted according to a protocol approved by Yale University’s Institutional Animal Care and Use Committee (protocol number 2022-20476). More details are depicted in Figure 2.

### Donor Preparation and Allograft Harvest

The donor pig was positioned supine on heat support, under isoflurane anesthesia (0.8%–2%). Following connection to monitoring equipment and IV fluid administration, the donor received prophylactic antibiotics (cefazolin) and analgesics (meloxicam, buprenorphine) alongside local anesthesia with bupivacaine at key surgical sites. Antiseptic preparation with povidone-iodine was applied to the head and neck.

A hemifacial flap was carefully marked on the donor pig’s face. Skin incisions were made along the brachiocephalic muscle and the neck, sparing the ear and eye, while advancing dissection above the periosteal plane in the nasal and fronto-parietal areas. The dissection proceeded superiorly to the mandible, preserving the external jugular vein. In the facial region, meticulous incisions were made around the auricular, eyelid, and oral areas, incorporating buccal mucosa and securing the salivary glands. Further, the submandibular gland was removed after ligating its vascular branches, and the facial artery and nerve were identified. The facial nerve was transected near the stylomastoid foramen, and the external carotid artery and external jugular vein served as the flap’s vascular pedicle. Following tissue elevations along the masseter muscle and excisions in the neck area, the sternomastoideus muscle was detached, exposing central vessels including the common carotid and its branches. Key arteries, such as the internal carotid, were ligated and transected.

The graft was perfused *in situ* until the recipient’s vasculature was ready. The donor’s central vessels were ligated, and the graft was flushed with heparin solution, followed by euthanasia with sodium pentobarbital as per established veterinary protocols.

### Recipient Preparation and Hemiface Graft Inset

The recipient pig was anesthetized and positioned supine with a 30° rotation to expose the dorsolateral side, allowing simultaneous preparation with the donor. A groin incision exposed the femoral vessels, isolated to allow anastomosis. A subcutaneous pocket was created from the groin to the dorsolateral abdominal wall, where the graft would be placed. The hemiface flap was inset dorsolaterally to facilitate immune monitoring.

Following ligation of the donor’s femoral vessels, the graft was prepared for anastomosis. Venous anastomoses were conducted with a vascular coupling device (2.5 mm size), while arterial anastomoses were sutured with 9-0 nylon. Once vascular patency was confirmed, the graft was secured in place with sutures to the abdominal wall muscles while the skin and mucosa paddle were exteriorized for monitoring. The groin incision was closed in layers and covered with a Tegaderm^®^ patch to prevent infection. Analgesia was administered via a fentanyl patch, and postoperative antibiotics were given. The recipient pig was monitored until full recovery.

The recipient pig recovered from the operation without any complications, exhibiting normal eating and drinking behavior and full mobility. Frequent monitoring revealed a viable flap, with the recipient site in the groin well-tolerated by the pig. After 24 h, the pig was euthanized according to protocol. These findings demonstrate the feasibility of our model for heterotopic hemiface vascularized composite allotransplantation in evaluating graft viability and immune response in a controlled and accessible site.

## Discussion

Our review highlights that specific experimental variables play a critical role in shaping long-term outcomes in swine VCA models. Graft composition emerged as a key determinant of immunogenicity and tolerance induction. Grafts that incorporate vascularized bone marrow (VBM) or osteomyocutaneous tissues consistently demonstrate enhanced tolerance induction, prolonged survival, and the establishment of mixed chimerism, compared to purely fasciocutaneous grafts. Multiple studies in large animal and rodent models show that inclusion of vascularized bone or bone marrow within the graft provides a continuous source of donor-derived hematopoietic stem cells, facilitating stable mixed chimerism and promoting donor-specific tolerance [47–49]. For example, in swine, protocols combining non-myeloablative conditioning, bone marrow infusion, and osteomyocutaneous VCA have achieved stable mixed chimerism and long-term graft survival across MHC barriers, with evidence of donor-specific hyporesponsiveness and regulatory T cell expansion. Similarly, in rodent models, VBM-containing grafts result in higher chimerism and longer allograft survival than non-osseous grafts, and removal of the VBM component abrogates tolerance [50, 51]. In contrast, purely fasciocutaneous or skin-only VCAs are more immunogenic and typically undergo earlier rejection, even under similar immunomodulatory protocols, and rarely achieve durable chimerism or tolerance [27, 49, 52]. The skin component remains the most challenging tissue for tolerance induction, and its rejection is accelerated in the absence of VBM [27, 38].

In addition, immunosuppressive regimens were found to vary widely, with tacrolimus serving as a cornerstone agent. Localized delivery of tacrolimus, such as via a hydrogel platform, has been shown to extend graft survival and reduce systemic toxicity compared to high-dose systemic regimens, as demonstrated by Fries et al. Low-dose tacrolimus hydrogel delayed acute rejection and was better tolerated, while high-dose regimens led to toxicity and poorer tolerability [35, 53–56]. Combination therapies, including tacrolimus with mycophenolate mofetil and methylprednisolone, are commonly used and have been associated with improved graft viability, bone remodeling, and minimal complications in large animal models, as described in systematic reviews and preclinical studies [57, 58]. Cellular therapies, such as mesenchymal stem cells (MSCs) and adipose-derived stem cells, have also been shown to modulate immune responses, promote regulatory T cell expansion, and extend rejection-free intervals, particularly when combined with short-course tacrolimus [59, 60].

Furthermore, monitoring strategies most commonly rely on clinical observation and histological grading of acute rejection, with relatively few studies employing serial biopsies or advanced immunophenotyping. The literature highlights that clinical assessment and histopathology—often using adaptations of the Banff criteria—are the mainstays for diagnosing and grading rejection, but there is a lack of standardized, reproducible protocols across studies, and serial or multimodal monitoring is not routine. Leonard et al. and Waldner et al. are exceptions to this general trend [58, 61, 62]. Leonard et al. correlated the presence of mixed chimerism with the histologic absence of acute rejection in swine VCA recipients, with tolerance and rejection-free survival documented up to postoperative day 504, integrating both chimerism analysis and histopathology for longitudinal monitoring. Waldner et al. specifically emphasized the correlation between clinical graft appearance and histological findings, using serial punch biopsies to confirm the progression of rejection in a swine myocutaneous VCA model [40, 63]. These variations in monitoring approaches highlight the need for standardized, reproducible protocols that integrate graft design, immunosuppressive regimens, and multimodal monitoring—including clinical, histological, and immunological parameters—to improve the translational value and comparability of swine VCA research.

Future research may also investigate swine VCA xenotransplants. Recent advancements in the field have introduced genetic engineering strategies to reduce the expression of swine xenogeneic antigens identifiable by human immunoglobulins, ultimately lessening the immunological rejection against xenotransplantation. For instance, Yoon et al. used CRISPR-CAS9 to target xeno-reactive genes GGTA1, CMAH, and B4GALNT2 from Jeju Native Pigs and develop triple-knockout pigs [64]. Genetically engineered pigs showed reduced expression of galactose-alpha-1,3-galactose and N-glycolylneuraminic acid, which have been previously identified as key drivers of xenorejection [65–67]. Overall, the removal of the three genes significantly reduced xenograft rejection and binding by human IgM and IgG antibodies [64]. Interestingly, another study used galactose-alpha-1,3-galactose/N-glycolylneuraminic acid double-knockout pig lungs that were perfused for up to 6 h with fresh heparinized human blood. The authors reported reduced antibody-mediated inflammation and activation of the coagulation cascade, as well as a delayed rise in pulmonary vascular resistance when compared to galactose-alpha-1,3-galactose single-knockout pig lungs [68]. Here, the authors highlighted that the additional N-glycolylneuraminic acid helps mediate the innate immune antigenicity in xenogenically perfused porcine lungs. Additional research has also underpinned the key role of the GGTA1, CMAH, β4GalNT2 and CIITA genes in activating human CD4^+^ T cells in 4-gene knockout pigs [69]. With these recent advancements on the clinical horizon, wild-type pigs may become increasingly obsolete, both scientifically and due to evolving regulatory standards. Vice versa, knockout pigs may serve as a valuable donor pool to catalyze the widespread clinical adoption of VCA and pave the way toward the first vascularized composite xenotransplantation (VCX) case [2].

The insights gained from this systematic review and our heterotopic hemiface model underscore the importance of swine models in particular in translating immunosuppressive strategies for human VCA. Our model’s ability to support serial biopsies of skin and mucosa provides a unique tool for examining the dynamics of immune tolerance and rejection, potentially improving clinical outcomes in patients undergoing complex tissue transplants. By optimizing immunosuppressive strategies to balance efficacy and safety, our model offers valuable guidance for refining VCA protocols, supporting the development of safer, more effective treatment paradigms in clinical transplantation.

### Limitations

This study is limited by the inherent heterogeneity of the swine models and experimental protocols reviewed, which complicates direct comparisons and generalizations across studies. The small sample sizes across the included studies reduce the generalizability of our findings and limit our ability to perform a robust quantitative meta-analysis. The rarity of VCA studies in swine also introduces potential publication bias, as studies with negative or inconclusive outcomes may be underreported.

Additionally, our heterotopic hemiface model, while valuable for serial biopsies, may not fully represent the complexity of vascular integration seen in orthotopic models, potentially limiting its direct applicability to specific clinical scenarios in VCA. Moreover, while the described heterotopic hemifacial VCA model was primarily designed to ensure surgical feasibility and facilitate serial mucosal and skin biopsies, we acknowledge the limitation that the transplanted mucosa is no longer located within its native anatomical environment. As such, it is exposed to non-physiological conditions, including external microbial flora and mechanical influences at the abdominal implantation site. These factors may affect local immune responses and limit the interpretability of biopsy-derived data with respect to natural mucosal immunity. Nevertheless, the model remains valuable for studying epithelial immune activation and early alloimmune events in a controlled and accessible setting, and it offers an important proof-of-concept for future refinements toward orthotopic models.

## Conclusion

Swine models have significantly advanced our understanding of VCA immunology through diverse composite grafts and immunomodulatory approaches. However, our review highlights a notable gap in models that specifically investigate facial VCAs, particularly those including the oral mucosa. Given the unique immunological environment of facial allografts, models such as the heterotopic hemiface transplant offer critical insights into immune mechanisms and provide a platform for refining targeted immunosuppressive strategies. By enabling serial biopsies and localized immune monitoring, this model addresses key challenges such as graft rejection and the systemic effects of immunosuppression. These advancements are essential for developing safer, more effective transplantation protocols, ultimately improving patient outcomes in facial VCA.

## Data Availability

The raw data supporting the conclusions of this article will be made available by the authors, without undue reservation.
