# Supplementary material for: Experimental Swine Models for Vascularized Composite Allotransplantation and Immunosuppression: A Systematic Review and Case Report of a Novel Heterotopic Hemifacial Swine Model
Source: Transpl Int. 2025 Jul 29;38:14520. doi: 10.3389/ti.2025.14520 (PMC12341719; doi:10.3389/ti.2025.14520)
Supplement: Supplementary file 2 [file Table1.docx]

**Supplement Table 1.** Search strings in PubMed/MEDLINE and Google Scholar databases with the respective number of search results.

| **Database** | **Search String** | **Results** |
| --- | --- | --- |
| PubMed/MEDLINE/Google Scholar | Swine[MeSH Terms] OR  Porcine[MeSH Terms] OR  Pig[MeSH Terms] OR  Sus Scrofa[MeSH Terms] OR  Miniature Swine[Title/Abstract] OR  Mini-Pig[Title/Abstract] OR  Mini Pig[Title/Abstract] OR  Porcine Model[Title/Abstract] OR  Porcine Species[Title/Abstract]  AND  Vascularized Composite Allotransplantation[MeSH Terms] OR  Vascularized Composite Allotransplantation[Title/Abstract] OR  Vascularized Composite Allograft[Title/Abstract] OR  Vascularized Composite Tissue Transplantation[Title/Abstract] OR  Vascularized Allogeneic Tissue[Title/Abstract] OR  Vascularized Allograft[Title/Abstract] OR  Composite Tissue Allotransplant[Title/Abstract] OR  Composite Tissue Allograft[Title/Abstract] OR  Composite Tissue Transplant[Title/Abstract]  AND  Immunosuppression[MeSH Terms] OR  Immune Suppression[Title/Abstract] OR  Immunomodulation[Title/Abstract] OR  Immunotolerance[Title/Abstract] OR  Immune Tolerance[Title/Abstract] OR  Immunoregulation[Title/Abstract] OR  Immune Evasion[Title/Abstract] OR  Immune Response[Title/Abstract] OR  Immune Inhibition[Title/Abstract] OR  Immune Tolerance Induction[Title/Abstract] OR  Immune Acceptance[Title/Abstract] | 6,040  (Filters applied: Full text, Case Reports, Clinical Study, Clinical Trial, Clinical Trial, Veterinary, Comparative Study, Controlled Clinical Trial, Multicenter Study, Observational Study, Observational Study, Veterinary, Randomized Controlled Trial, Veterinary, English, Other Animals) |
