# Supplementary material for: Experimental Swine Models for Vascularized Composite Allotransplantation and Immunosuppression: A Systematic Review and Case Report of a Novel Heterotopic Hemifacial Swine Model
Source: Transpl Int. 2025 Jul 29;38:14520. doi: 10.3389/ti.2025.14520 (PMC12341719; doi:10.3389/ti.2025.14520)
Supplement: Supplementary file 3 [file Table2.docx]

| **Supplement Table 2.** Risk of bias assessment by type of bias according to SYRCLE's bias risk tool | | | | | |  |
| --- | --- | --- | --- | --- | --- | --- |
| **Bias** | Selection | Performance | Detection | Attrition | Reporting | Other |
| **Study** |  |  |  |  |  |  |
| Barone et al., 2015 | Low | Unclear | Unclear | Low | Low | Low |
| Berkane et al., 2024 | Low | Unclear | Low | Low | Low | Low |
| Blades et al., 2024 | Low | Unclear | Unclear | Low | Low | Low |
| Elgendy et al., 2022 | Low | Unclear | Low | Low | Low | Low |
| Fries et al., 2019 | Low | Unclear | Low | Low | Low | Low |
| Ibrahim et al., 2013 | Unclear | Unclear | Low | Low | Low | Low |
| Kim et al., 2018 | Low | Unclear | Low | Low | Low | Low |
| Kotsougiani et al., 2016 | Low | Unclear | Low | Low | Low | Low |
| Kuo et al., 2009 | Low | Unclear | Low | Low | Low | Low |
| Kuo et al., 2009 | Low | Unclear | Low | Low | Low | Low |
| Kuo et al., 2011 | Low | Unclear | Unclear | Low | Low | Low |
| Kuo et al., 2017 | Low | Unclear | Low | Low | Low | Low |
| Leonard et al., 2014 | Low | Unclear | Low | Low | Low | Low |
| Mathes et al., 2014 | Low | Unclear | Low | Low | Low | Low |
| Park et al., 2016 | Low | Unclear | Low | Low | Low | Low |
| Shanmugarajah et al., 2016 | Low | Unclear | Low | Low | Low | Low |
| Tratnig-Frankl et al, 2024 | Low | Unclear | Low | Low | Low | Low |
| Wachtman et al., 2011 | Low | Unclear | Low | Low | Low | Low |
| Waldner et al., 2020 | Low | Unclear | Low | Low | Low | Low |
| Wang et al., 2015 | Low | Unclear | Low | Low | Low | Low |
| Wu et al., 2016 | Unclear | Unclear | Low | Low | Low | Low |
| Zhang et al., 2024 | Low | Unclear | Low | Low | Low | Low |
